# Preliminary Assessment of the Mucosal Toxicity of Tea Tree (*Melaleuca alternifolia*) and Rosemary (*Rosmarinus officinalis*) Essential Oils on Novel Porcine Uterus Models

**DOI:** 10.3390/ijms21093350

**Published:** 2020-05-09

**Authors:** Martina Bertocchi, Antonella Rigillo, Alberto Elmi, Domenico Ventrella, Camilla Aniballi, Diana G. Scorpio, Maurizio Scozzoli, Giuliano Bettini, Monica Forni, Maria Laura Bacci

**Affiliations:** 1 Department of Veterinary Medical Sciences, University of Bologna, 40064 Ozzano dell’Emilia (BO), Italy; martina.bertocchi3@unibo.it (M.B.); antonella.rigillo2@unibo.it (A.R.); domenico.ventrella2@unibo.it (D.V.); camilla.aniballi2@unibo.it (C.A.); diana.scorpio@nih.gov (D.G.S.); giuliano.bettini@unibo.it (G.B.); monica.forni@unibo.it (M.F.); marialaura.bacci@unibo.it (M.L.B.); 2 Vaccine Research Center, National Institute of Allergy and Infectious Diseases, National Institutes of Health, Bethesda, MD 20892, USA; 3 Freelance Veterinarian, 47122 Forlì (FC), Italy; maurizio@greenvet.com

**Keywords:** swine reproduction, uterine mucosa, in vitro model, ex vivo model, essential oil, *Melaleuca alternifolia*, *Rosmarinus officinalis*

## Abstract

Antimicrobial resistance, an ever-growing global crisis, is strongly linked to the swine production industry. In previous studies, *Melaleuca* *alternifolia* and *Rosmarinus* *officinalis* essential oils have been evaluated for toxicity on porcine spermatozoa and for antimicrobial capabilities in artificial insemination doses, with the future perspective of their use as antibiotic alternatives. The aim of the present research was to develop and validate in vitro and ex vivo models of porcine uterine mucosa for the evaluation of mucosal toxicity of essential oils. The in vitro model assessed the toxicity of a wider range of concentrations of both essential oils (from 0.2 to 500 mg/mL) on sections of uterine tissue, while the ex vivo model was achieved by filling the uterine horns. The damage induced by the oils was assessed by Evans Blue (EB) permeability assay and histologically. The expression of ZO-1, a protein involved in the composition of tight junctions, was assessed through immunohistochemical and immunofluorescence analysis. The results showed that low concentrations (0.2–0.4 mg/mL) of both essential oils, already identified as non-spermicidal but still antimicrobial, did not alter the structure and permeability of the swine uterine mucosa. Overall, these findings strengthen the hypothesis of a safe use of essential oils in inseminating doses of boar to replace antibiotics.

## 1. Introduction

Antimicrobial resistance represents a global crisis strongly affecting public health, animal health & welfare and food security, and an integrated “One Health” response is essential to avoid this universal threat [1]. The use of antibiotics in food-producing animals plays a pivotal role as major driving force behind the rise in the prevalence of antibiotic resistance [2,3]. As reported for many species, antimicrobial resistance is also increasing within the swine production industry [4]. Artificial insemination (AI) is the most common breeding procedure in pigs, used to fecundate more than 90% of the sows in the world’s primary pork-producing countries [5,6]. Liquid semen refrigerated at 16 ± 1 °C, and preserved up to seven days in long-term extenders, is routinely used in pig farms and breeding centers around the world. To control bacterial growth, antimicrobial agents are essential components of semen extenders [5]. In the European Union, these regulations are outlined in Council Directive 90/429/EEC [7]. A major concern is the generation of antibiotic-resistant bacterial strains in AI stations, triggered by the intense use of antibiotics in semen extenders. Seventy to 90% of the volume of the semen dose is retrograde eliminated from the uterus after AI [8], thus considerable amounts of antibiotic and potentially resistant bacteria enter the manure. The progressive ban of certain antibiotics for veterinary use [9], reinforces the need for alternative antimicrobial strategies in boar semen extenders [5]. During the last decades, physical approaches were developed such as bacteria removal by colloid centrifugation [10] and microfiltration of seminal plasma [11], or the use of antimicrobial peptides [12,13]. At the same time, natural compounds, and in particular essential oils (EOs) were evaluated on semen of different species for their antioxidant and antimicrobial proprieties [14,15,16,17].

Despite containing two or three main components at a level of 20%–70%, EOs are very complex mixtures of mainly terpenes, terpenoids, and phenylpropanoids [18]. The mechanisms of action of EOs depend on their chemical composition, and their antimicrobial activity is not attributable to a unique mechanism but is instead a cascade of reactions involving the entire bacterial cell [19]. The essential oil derived from *Melaleuca alternifolia* (Maiden and Betche) Cheel., an Australian native plant belonging to the *Myrtaceae* family, is known as tea tree oil (TTO). Its composition is regulated by an international standard for “Oil of Melaleuca—terpinen-4-ol type,” which sets maxima and/or minima for 14 components of the oil [20]. When the TTO contains 30% to 40% of terpinen-4-ol, identified as the most effective antimicrobial component, it is referred to as terpinen-4-ol chemotype [21,22]. TTO possesses antimicrobial properties also against multi-drug resistant Gram-negative bacteria and methicillin-resistant *Staphylococcus aureus* [23,24]. *Rosmarinus officinalis* Linnaeus (1753, *Lamiaceae*), is a plant native to the Mediterranean area, but cultivated worldwide, known for its nutritional value and pharmacological properties (e.g., anti-inflammatory, antiparasitic and antimicrobial) [25,26]. In the food industry, it is used as flavoring and preservative agent due to its antioxidant and antimicrobial potential [26,27]. Rosemary EO is usually classified into chemotypes according to its most abundant chemical component, responsible for the majority of its pharmacological activities: 1,8-cineole, camphor, and α-pinene [26,28]. 

Regarding the porcine reproduction field, both *M. alternifolia* and *R. officinalis* EOs have already been evaluated for spermatozoa toxicity [29,30] and for their antimicrobial capabilities in AI doses [31]. They exhibit concentration-dependent effects, with different patterns of morphofunctional damage on spermatozoa, but concentrations lower than 0.6 mg/mL were proved as non-cytotoxic [29,30]. Both *M. alternifolia* and *R. officinalis* EOs, at the concentration of 0.4 mg/mL, were capable of exerting effects similar to those of ampicillin on the liquid phase of AI doses added with a standardized quantity of *E. coli* and stored at 16 ± 1 °C up to 5 days [31]. On the bases of such studies, both *M. alternifolia* and *R. officinalis* EOs have proved to be potentially useful as antimicrobial agents for reproductive biotechnologies, but studies regarding their potential effects on sows are lacking. This gap needs to be filled before being able to safely move on to in vivo reproduction trials.

Therefore, the aim of the present research was to develop and validate an in vitro and an ex vivo model of porcine uterine mucosa for the evaluation of the potential effects of *M. alternifolia* and *R. officinalis* EOs on the endometrial tissue.

## 2. Results

The organs arrived at the laboratory in less than 2 h, thus within the period during which mucosal tissue remains vital and retains its barrier function [32]. All the uteri (*n* = 8) included in the experimental protocol did not show any macroscopically visible alteration imputable to pre-existing conditions. The follicular phase was confirmed by direct visualization of gonads before ablation for the experimental purposes, and all uteri were in the follicular phase (proestrus).

### 2.1. Quantitative Evaluation of Uterine Mucosa Permeability to Evans Blue (EB) Dye

**Figure 1:**
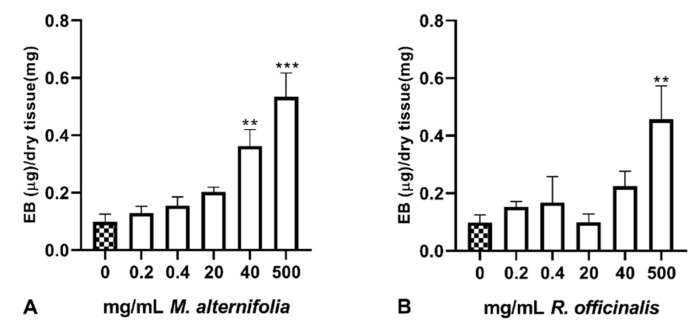
Quantitative evaluation of uterine mucosal damage induced by essential oils (EOs) by means of Evans Blue (EB) permeability assay. (**A**) *Melaleuca alternifolia* (**B**) *Rosmarinus officinalis.* Data are expressed as mean ± standard error of the mean (*n* = 8). 0 mg/mL represents the control sample (only emulsifiers). ** *p* < 0.01, *** *p* < 0.001.

The Evans Blue (EB) dye is known to quantitatively bind albumin both in vivo and in vitro. This property has been widely used to quantify protein leakage as an indicator of increased epithelial permeability, thus mucosal injury [33,34,35]. In order to quantify the uterine mucosal damage induced by *M. alternifolia* and *R. officinalis* EOs, five different concentrations for each EOs were evaluated. The output of the two-way analysis of variance (ANOVA), performed to evaluate the effect of the treatments (Ma VS Ro) and concentrations, indicated no differences between the two EOs (*p* = 0.101). Since, on the other hand, concentrations were statistically effective (*p* < 0.001), a one-way ANOVA for each oil was performed followed by a Dunnet test. Data regarding the quantity of EB dye absorbed by the different tissue samples and statistical outcomes are reported in Figure 1.

When compared to the control samples, Ma EO induced a dose-dependent increase in EB quantity that became statistically significant at the concentrations of 40 and 500 mg/mL (*p* < 0.01 and *p* < 0.001, respectively). On the other hand, Ro EO only determined a statistical difference at the highest concentration (positive control) (500 mg/mL, *p* < 0.01).

### 2.2. Histology

**Figure 2:**
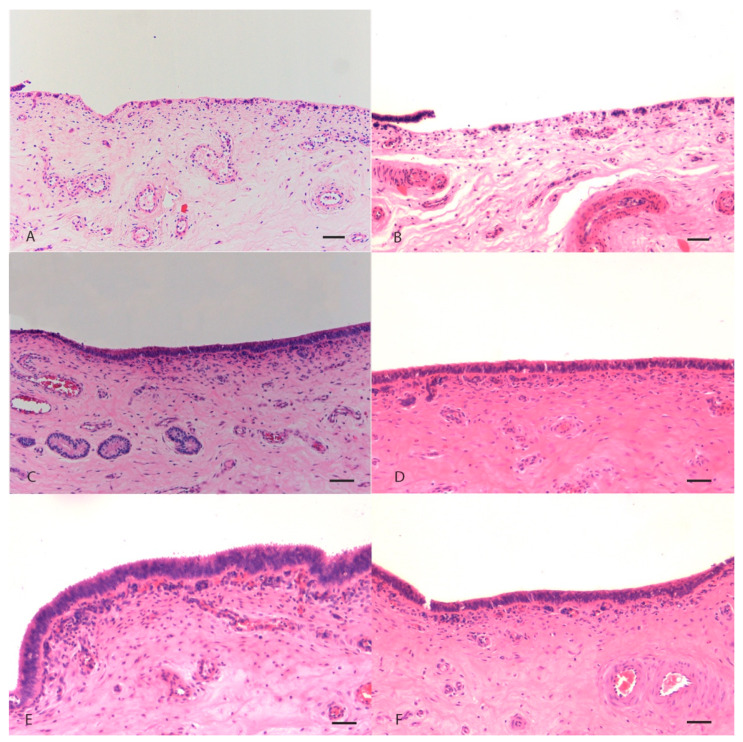
Histological sections of porcine uterine mucosa incubated in vitro with *Melaleuca alternifolia* and *Rosmarinus officinalis* essential oils. Tissue incubated with concentration of 500 mg/mL (positive control) Ma (**A**) and Ro (**B**); there is diffuse detachment and loss of mucosal epithelium. Tissues incubated with 0.4 mg/mL Ma (**C**), 0.4 mg/mL Ro (**D**), 0.2 mg/mL Ma (**E**) and 0.2 mg/mL Ro (**F**) did not show any mucosal damage. Hematoxylin-eosin; 100×. Bar = 50 µm.

**Figure 3:**
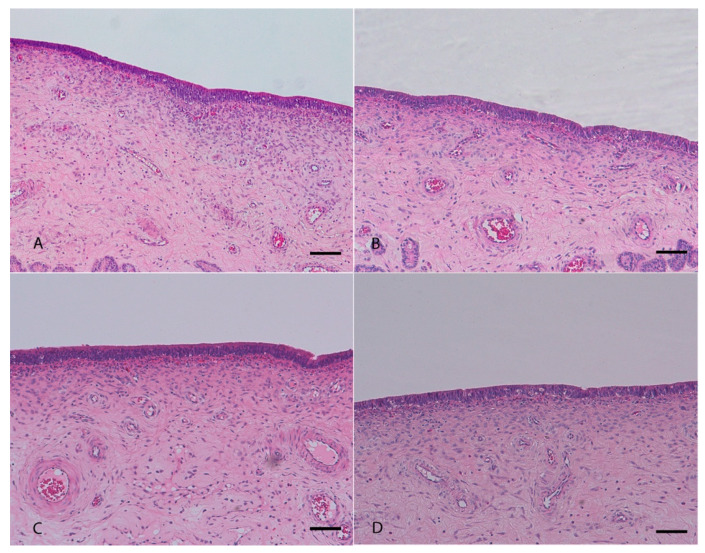
Histological sections of porcine uterine mucosa incubated ex vivo with *Melaleuca alternifolia* and *Rosmarinus officinalis* essential oils. Tissues incubated with 0.4 mg/mL Ma (**A**), 0.4 mg/mL Ro (**B**), 0.2 mg/mL Ma (**C**) and 0.2 mg/mL Ro (**D**) did not show any mucosal damage. Hematoxylin-eosin; 100×. Bar = 50 µm.

Histopathological examination of uterine mucosa showed, as main feature, a partial or complete detachment of the epithelial layer and, less seldom, thinning of pseudostratified prismatic cells to flattened single layer epithelium in damaged to non-damaged transition areas. Severe mucosal damage was only found in positive control samples treated with the highest concentration (500 mg/mL) of both *M. alternifolia* and *R. officinalis* EOs (Figure 2A,B). A moderate mucosal damage was also present in samples treated with 40 mg/mL of *M. alternifolia* EO. No damage was present in tissues incubated with 20 mg/mL, 0.4 mg/mL and 0.2 mg/mL concentrations of both EOs (Figure 2) including in negative control with emulsifiers only (Figure S1A). Results were comparable for both in vitro and ex vivo assays (Figure 3).

### 2.3. Immunohistochemistry and Immunofluorescence

**Figure 4:**
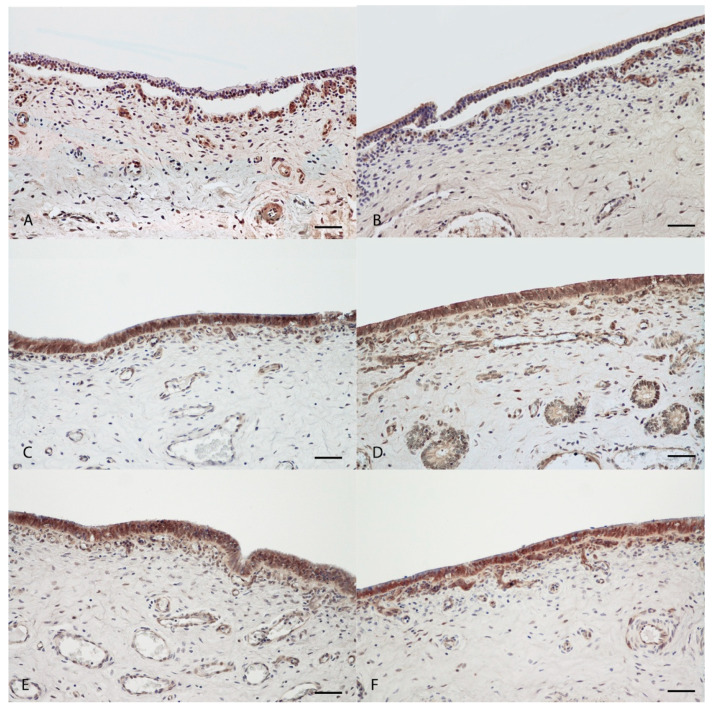
Immunohistochemical assessment of ZO-1 expression in porcine uterine mucosa incubated in vitro with *Melaleuca alternifolia* and *Rosmarinus officinalis* essential oils. Tissue incubated with concentration of 500 mg/mL (positive control) Ma (**A**) show lacking antibody expression within mucosal epithelium; tissue incubated with concentration of 500 mg/mL (positive control) Ro (**B**) show mild and rare positive expression of epithelial cells. Tissues incubated with 0.4 mg/mL (**C**, Ma and **D**, Ro) and 0.2 mg/mL (**E**, Ma and **F**, Ro) concentrations of both oils revealed intense and diffuse cytoplasmic expression of ZO-1 protein within mucosal epithelium. 200×. Bar = 50 µm.

**Figure 5:**
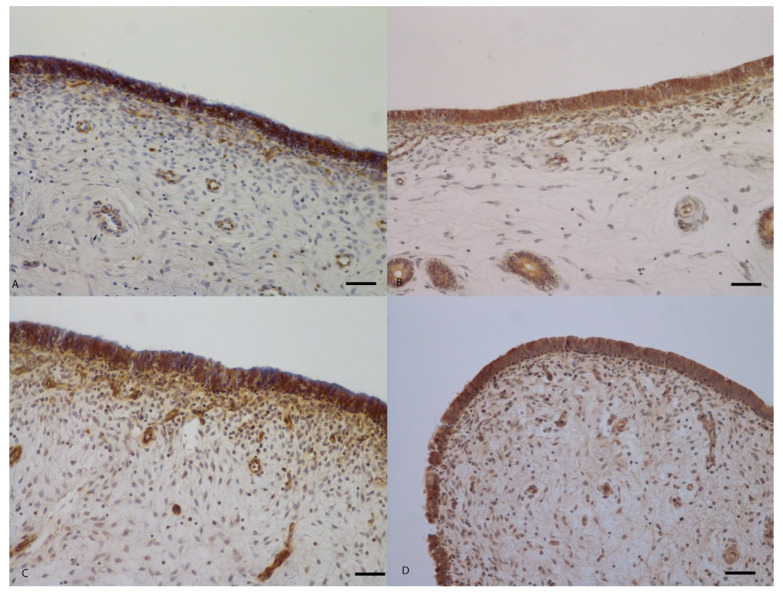
Immunohistochemical assessment of ZO-1 expression in porcine uterine mucosa incubated ex vivo with *Melaleuca alternifolia* and *Rosmarinus officinalis* essential oils. Tissues incubated with 0.4 mg/mL (**A**, Ma and **B**, Ro) and 0.2 mg/mL (**C**, Ma and **D**, Ro) concentrations of both oils revealed intense and diffuse cytoplasmic expression of ZO-1 protein within mucosal epithelium. 200×. Bar = 50 µm.

**Figure 6:**
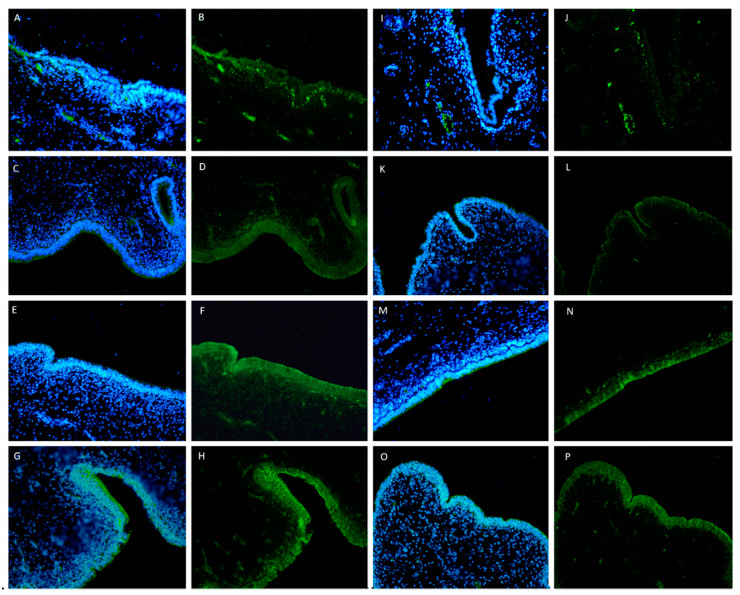
Immunofluorescence assessment of ZO-1 expression (green) in porcine uterine mucosa incubated in vitro and ex vivo with *Melaleuca alternifolia* and *Rosmarinus officinalis* essential oils. Tissue incubated with concentration of 500 mg/mL (positive control) Ma (**A**,**B**) and Ro (**I**,**J**) show mild to absent antibody expression within mucosal epithelium. Tissues incubated with 0.4 mg/mL (**C**,**D**, Ma and **K**,**L**, Ro) and 0.2 mg/mL (**E**,**F** Ma and **M**,**N**, Ro) concentrations of both oils revealed intense and diffuse cytoplasmic expression of ZO-1 protein within mucosal epithelium, comparable with control samples (**G**,**H**,**O**,**P**). Nuclei staining with Hoechst (blue); merge (**A**,**C**,**E**,**G**,**I**,**K**,**M**,**O**). ZO-1 expression (green) (**B**,**D**,**F**,**H**,**J**,**L**,**N**,**P**). Magnification: 200×.

Immunohistochemical analyses revealed mild to absent expression of ZO-1 within detached mucosal epithelium of positive control samples incubated with 500 mg/mL of both EOs (Figure 4A,B), while the expression was intense and present in epithelial cells of samples treated with all other concentrations of both EOs, regarding in vitro and ex vivo assays (Figure 4 and Figure 5) including in negative control with emulsifiers only (Figure S1B). Upon immunofluorescence, expression of ZO-1 was comparably mild to absent in epithelial cells of uterine samples treated with 500 mg/mL concentration (positive control) of both EOs (Figure 6A,B,I,J, Ma and Ro, respectively) and moderately intense but diffused in those treated with 40 mg/mL concentration of both EOs. The 0.4 and 0.2 mg/mL concentrations, in both in vitro and ex vivo assays, did not show any remarkable alterations in ZO-1 expression when compared to control samples (Figure 6). 

## 3. Discussion

The challenges connected to the spread of antibiotic resistance associated with the use of antibiotics in livestock and in particular in swine-producing farms are very complex and have multifaceted effects not only on animals, but also on humans and the environment [36]. Given the increasing need to reduce the use of antibiotics, Elmi and colleagues proposed the use of essential oils of *Melaleuca alternifolia* and *Rosmarinus officinalis* as antimicrobial agents in porcine inseminating doses, as an alternative to the antibiotics used in semen extenders. The published studies reported that relatively low concentrations of both EOs (0.2 and 0.4 mg/mL) were non-cytotoxic on porcine spermatozoa [29,30] and capable of exerting similar effects to those of ampicillin on swine AI doses [31]. Despite the basis laid by these studies, it is necessary to evaluate the potential toxicity of these EOs on the female reproductive system before safely proposing their use for in vivo reproduction. In such scenario, the present work aimed at the development and validation of in vitro and ex vivo models of swine uterine mucosa to study mucosal cytotoxicity.

Some ex vivo models using porcine vagina were developed for studying permeability and pathogenesis in mucosa [32,37] or to assess drug permeation from mucoadhesive and colloidal pharmaceutical systems [38]. Other models already developed include a long-term perfused uterus model [39] and an ex vivo one used to investigate fertilization within the oviductal environment [40]. Nonetheless, to the best of the authors’ knowledge, this is the first work reporting in vitro and ex vivo models of porcine uterine mucosa developed to assess essential oils toxicity. In order to make the scenario as close as possible to the in vivo insemination situation, cyclic sow organs in follicular phase were used. Moreover, to standardize both the in vitro and ex vivo models, we decided to set incubation time to 1 h. Indeed, during in vivo AI, the most common swine reproductive technology, the physiological gelatinous plug produced by the last part of the boar ejaculate that would “close” the cervix to prevent backflow, is obviously lacking. This makes for a rapid elimination of the inserted fluid, with more than 80% of it being leaked within 2 h. Moreover, in in vivo conditions, the AI dose is be immediately diluted by the mucosal secretion within the uterus [8,41,42]. This is why we felt confident performing the treatment for 1 h, in a closed system where both backflow and dilution would be impossible. Our model may represent the worst case scenario, since potential damaging compound are left in direct contact with the mucosa for a very long time.

The results of the in vitro model suggest that both essential oils induced severe damage to the uterine mucosa when used at the highest concentration (500 mg/mL). The decision to use an extremely high concentration of essential oils as positive toxic controls was mainly driven by the absence in the current literature, to the best of the authors’ knowledge, of toxicity evaluations on porcine uterine mucosa. In literature, there are not experimental protocols assessing and describing toxic effect of compounds on such tissues in ex vivo or in vitro settings reported, where only mucosal disruption can be expected since systemic responses such as inflammation are impossible to achieve. Nonetheless, literature has only described how essential oils are toxic on different cell populations. Hammer et al. [43] reviewed the toxicity of *M. alternifolia* EOs, indicating how this phytocomplex can exert negative effects at concentrations way lower than 500 mg/mL: concentrations ranging from 0.02 to 2.7 mg/mL were capable of reducing the growth of cells by 50% (IC_50_) for HeLa, K562, CTVR-1, Molt-4, Hep G2, HL-60, fibroblast and epithelial cells. Also, regarding *R. officinalis* EOs, cytotoxicity was reported for extremely low concentrations, with a IC_50_ of 0.25 mg/mL on cancer cell lines and way lower in other cells [25]. Therefore, on the basis of the literature analysis, we felt comfortable in using extremely high concentrations of essential oils as positive controls. 

Evans Blue permeability assay, allowing to quantify the damage induced by the EOs, showed dose-dependent effects of *M. alternifolia* EO that became significant only at the higher concentrations (40 and 500 mg/mL). This result is in line with the available literature as, despite *M. alternifolia* essential oil being commonly used, toxic effects have been reported at higher concentrations [43]. Furthermore, Ma EO and its major component terpinen-4-ol showed significant cytotoxic effects and anti-proliferative activity against murine tumor cell lines [44] and human non-small cell lung cancer cells [45].

The results were confirmed by histological analyses: indeed a detachment of the epithelium, locally extended to more than 70% of the section, was observed in all positive control samples treated with the highest concentration of both essential oils. At 40 mg/mL, *M. alternifolia* induced moderate damage to the uterine mucosa, with a multifocal detachment of the epithelium while the Ro EO caused milder damage with fewer detachment points of the epithelium. At the lower concentrations, both essential oils did not modify the tissue architecture that was comparable to the control, thus confirming the quantitative results obtained with the EB permeability assay. In general, Ro EO appeared less toxic than *M. alternifolia* EO to the porcine uterine mucosa, confirming the previously reported relative lower toxicity of *R. officinalis* EO [26]. 

Zonula occludens 1 (ZO-1), a member of the membrane-associated guanylate kinase protein family, serves as a scaffold to organize transmembrane tight junction (TJ) proteins and plays a key role in retaining barrier integrity and TJs during pathological injury [46,47]. Its distribution was examined as a marker protein for tight junctional complexes. In control samples, the immunostaining pattern of ZO-1 was typical of tight junctional complexes of epithelial cells. With immunofluorescence (IF) analysis in particular, it was possible to observe that ZO-1 was localized in a discrete apical region of the lateral membrane in the epithelium. These results confirmed what was previously observed by Cencič and colleagues on samples of the uterus of cyclic sows [48]. This immunostaining pattern of ZO-1 was present also in all samples treated with a low concentration of both EOs. On the other hand, in the positive control samples at the highest concentration, a weak or absent expression of ZO-1 was observed on the epithelium, which is detached from the basement membrane. In the gut, tight junctions play a key role in permeability and barrier function, in particular, external stimuli can alter this balance and determinate a dysregulation of tight junction permeability and loss the barrier function [49]. This mechanism has been reported for many bowel disease [50,51]. In piglets, a decrease in ZO-1 protein in the intestinal tissues of animals treated with deoxynivalenol, highly toxic to animals and humans, was reported. These results indicated that a toxic compound can induce intestinal damage altering mucosa permeability [52]. A profound alteration in TJ proteins and a decrease of ZO-1 of uterine epithelial cells, due to perinatal exposure of bisphenol-A, was observed in the early stage of pregnancy in rats [53]. On the basis of this studies, we hypothesized that high concentrations (500 mg/mL) of *M. alternifolia* and *R. officinalis* essential oils may operate as a toxic, altering the permeability of the uterine mucosa and reducing the expression of ZO-1. However, this mechanism was not observed at low concentrations of both OEs, confirming that these essential oils can have a very different function based on concentration in porcine uterine mucosa.

## 4. Materials and Methods 

The EOs of *M. alternifolia* (Ma) and *R. officinalis* (Ro) used in the present study were supplied by APA-CT S.r.l. (Via Sacco Nicola, 22, 47122, Forlì, Italy) and their chemo-characterization was previously reported by the authors in the aforementioned toxicity studies [29,30]. For experimental purposes, the EOs were reconstituted in 0.5% dimethylsulfoxide (DMSO) with Tween 80 (0.02% *v*/*v*) to facilitate diffusion in water-based solutions [54]. The experimental treatments were prepared by diluting the reconstituted EOs in Swine Fertilization Medium (SFM) to obtain 5 different concentrations: 0.2, 0.4, 20, 40 and 500 mg/mL, the latter being used as positive control since it was extremely high. The SFM extender was prepared as described by Fantinati et al. [55], without any antibiotic. For each experiment, negative control samples were realized by only adding the emulsifiers (DMSO 0.5% *v*/*v* and Tween 80 0.02% *v*/*v* [54]) to SFM extender. All reagents, unless otherwise specified, were purchased from Sigma-Aldrich (Saint Louis, MO, USA).

### 4.1. Experimental Design

Female reproductive tracts (*n* = 8) were collected from adult sow at a local slaughterhouse from commercially available European breed pigs. After collection, organs were transferred to the physiology labs of the Department of Veterinary Medical Sciences of the University of Bologna within 2 h. Upon arrival, each organ was thoroughly rinsed with tap water and then isolated from other contiguous organs (vulva, anus, bladder, vagina and cervix). Only uteri without visible alterations were used for the experimental purposes. The estrous cycle stage was determined by observing corpora lutea and follicles on the ovaries. All 8 uteri were used for both in vitro and ex vivo models.

### 4.2. In Vitro Model 

A tract of 15 cm was isolated from the left horn, 10 cm cranially the uterus bifurcation. The tract was longitudinally opened and washed twice by dipping in sterile saline solution (NaCl 0.9%). Eleven biopsies (~3 cm × 3 cm) were cut from each section and placed individually in petri dishes. 100 µL of each treatment, included positive control (500 mg/mL) and negative control with SFM and only emulsifiers, were dispensed on the surface of each fragment, assuring entire surface covering. The sections were then incubated in a 5% CO_2_ atmosphere, 100% relative humidity, at 38.5 °C for 1 h. After incubation, each section was washed by dipping in Dulbecco’s phosphate-buffered saline (DPBS) (Gibco-Life technologies Carlsbad, CA, USA) and split into two portions. One was used for histological, immunohistochemical (IHC) and immunofluorescence (IF) analyses. The other was used to evaluate the mucosal damage with an EB permeability assay.

### 4.3. Ex Vivo Model 

**Figure 7:**
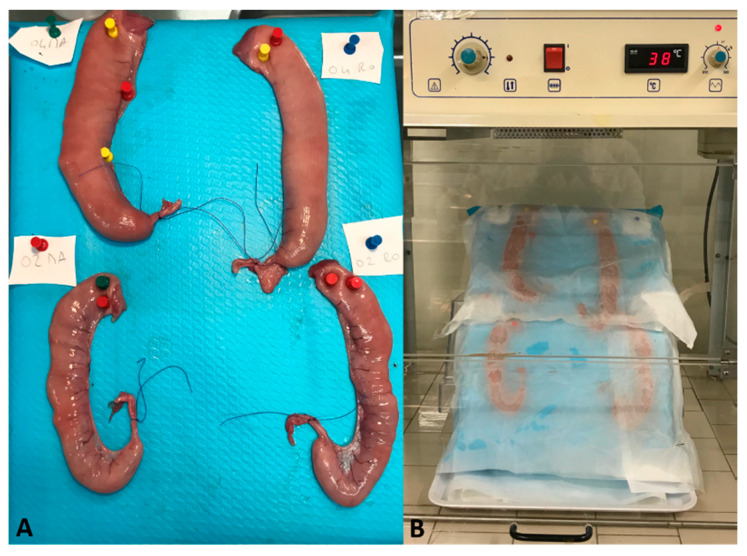
Porcine uterus ex vivo model. Uterine horns pinned on the support and filled with treatments (**A**) Experimental set up in the thermostatic hood (**B**).

After removing the ovaries and the infundibulum, each uterine horn was cut at 20 cm from the utero-tubal junction. The uterine oviduct was cannulated with an intravenous (IV) catheter (G20) then the tube and the uterine horn tract were washed with 10 mL of sterile saline solution. The uterine horns were pinned upright on an oblique polystyrene support with an inclination of approximately 45 degrees. The treatment was performed filling each horn with 10 mL of one type of solution (0.2 mg/mL Ma; 0.4 mg/mL Ma; 0.2 mg/mL Ro; 0.4 mg/mL Ro). The uterine horns were incubated for 1 h at 38.5 °C in thermostatic hood (Climatic Hood 810; ASAL s.r.l., Cernusco sul Naviglio, Italy), as reported in Figure 7. Organs were kept moist outside by means of wet tissue paper. After incubation, each uterine horn was emptied, washed with DPBS and a section of 3 cm × 3 cm was sampled for histological, immunohistochemical (IHC) and immunofluorescence (IF) analyses.

### 4.4. Evans Blue Permeability Assay 

In order to evaluate the mucosal damage induced by treatment with Ma and Ro OEs, an EB permeability assay was performed. 50 µL of Evans Blue solution (10 mg/mL in physiological saline solution) were placed on the mucosal layer of each fragment from the in vitro assay. After 10 min, samples were washed twice in saline solution (NaCl 0.9%). Tissue fragments were then dried at 37 °C in a thermostatic hood for 1 h. Afterward, the mucosa layer was isolated under a stereomicroscope and immediately weighed. Each mucosa sample was placed in 3 mL of formamide and incubated for 48 h at 50 °C in order to extract EB dye. Colorimetric measurements were performed using a microplate reader (Infinite^®^ F50, Tecan, Trading AG, Switzerland) at the maximum absorption for EB (620 nm). Each sample was analyzed in triplicate. EB quantity extracted for each tissue was obtained taking into account a calibration curve (0.025, 0.25, 0.5, 1.25, 5, 12.5, 25 μg/mL EB in formamide) and normalized on sample weight (in milligrams).

### 4.5. Histological, Immunohistochemical and Immunofluorescence Analyses

Tissue samples were fixed in 4% formaldehyde solution for at least 24 h. Of each sample, two serial sections 3 mm thick were trimmed from the central part of the fragment and embedded in paraffin blocks; two serial sections 3 µm thick were cut and routinely stained for hematoxylin-eosin. On the basis of the histopathological features, the mucosal damage was evaluated as the presence of epithelial detachment or thinning in more than 70% of the sections.

For immunohistochemical analysis, 3 µm-thick sections were immunolabeled with primary polyclonal anti-rabbit ZO-1 (Zonula occludens 1) antibody (ZO-1 polyclonal antibody, clone 61-7300, Invitrogen, Carlsbad, California, USA) with validated reactivity in porcine heart [56]. Endogenous peroxidases were blocked by incubation for 15 min with 3% hydrogen peroxide in methanol. For antigen retrieval, slides were incubated for 20 min at 37 °C with protease XIV enzyme (protease from *Streptomyces griseus,* P5147, Sigma-Aldrich, St Louis, Missouri, USA). Sections were then incubated overnight at 4 °C in a humid chamber at 1:200 dilution. Sites of primary antibody binding were identified by incubation with appropriate biotinylated secondary antibody (Code No. E 0432, Dako—Glostrup, Danimarca) at a dilution of 1:200 in blocking solution. Sections were incubated with a commercial streptavidin-biotin-peroxidase kit (Vectastain Elite ABC Kit, Vector Laboratories, Burlingame, CA, USA) for 30 min; 3,3’-diaminobenzidine (DAB, Diagnostic BioSystems, Pleasanton, CA, USA) was used as chromogen. Sections were ultimately counterstained with Papanicolaou’s hematoxylin, dehydrated and mounted. Swine heart was used as positive controls [56]. Negative controls were obtained by omitting the primary antibody. The expression was evaluated in terms of the presence and intensity of intracytoplasmic positive (brown) staining in epithelial cell of the uterine mucosa. 

For immunofluorescence analysis, sections were incubated with the same primary antibody at the same concentration overnight at 4 °C. Sites of primary antibody binding were identified by incubation with conjugated secondary antibody Alexa fluor 488 (Goat anti-Rabbit IgG (H+L) Highly Cross-Adsorbed Secondary Antibody, Alexa Fluor Plus 488, clone A32731 Invitrogen, Carlsbad, California, USA) diluted 1:500 in 2% BSA, for one hour at room temperature, and then 10 min with Hoechst 33342 (Invitrogen, Carlsbad, California, USA) (stock solution 10,000 µg/mL) diluted 1:1000 in phosphate-buffered saline (PBS), for nuclear labeling. Slides were ultimately mounted and analyzed with digital image software ACT-2U associated with an Eclipse E600 epifluorescence microscope equipped with a Nikon digital camera (Nikon, Tokyo, Japan). 

### 4.6. Statistical Analyses

The statistical analyses were performed using the software GraphPad Prism v.8 (GraphPad Software Inc., San Diego, CA, USA). Descriptive statistics were calculated and expressed as means and standard error of the mean. Normal distribution was assessed by means of the Shapiro–Wilk test (*p* < 0.05). To evaluate differences between EOs and concentrations, two-way ANOVA was performed (significance level set at 0.05). To evaluate differences between the treated samples and the control one, a one-way ANOVA followed by the Dunnett post-hoc test was performed (significance level set at 0.05). 

## 5. Conclusions

In conclusion, the proposed in vitro and ex vivo model of swine uterine mucosa to evaluate the effects of essential oils seems to be capable of providing robust and sensitive results. Furthermore, these models proved to be also repeatable using uteri of sows from the slaughterhouse, in full accordance with the principles of the 3Rs. Overall, the results of the present study shown that low concentrations (0.2 and 0.4 mg/mL) of essential oils of *M. alternifolia* and *R. officinalis* are not toxic, and do not alter the structure and permeability of the swine uterine mucosa. The present research is part of a wide project aimed to use essential oils as antimicrobial agents in reproductive biotechnology. After showing that these essential oils, at the studied concentrations, do not alter the functions of the porcine spermatozoa, they also show antibacterial activities comparable to a common antibiotic in vitro, and not altering the uterine mucosa of sow, and the following step will be to use them in vivo in porcine inseminating doses as alternative to antibiotics.

## Supplementary Materials

Supplementary materials can be found at https://www.mdpi.com/1422-0067/21/9/3350/s1. Figure S1. Histologic appearance and immunohistochemical expression of ZO-1 of negative control tissues of porcine uterine mucosa.

## Abbreviations

AI	Artificial insemination
EOs	Essential oils
TTO	Tea tree oil
Ma	M. alternifolia
Ro	R. officinalis
3Rs	Replacement, Reduction and Refinement
EB	Evans Blue
DMSO	Dimethylsulfoxide
SFM	Swine Fertilization Medium
IHC	Immunohistochemical
IF	Immunofluorescence
ZO-1	Zonula occludens 1
BSA	Bovine serum albumin
PBS	Phosphate-buffered saline
DPBS	Dulbecco’s phosphate-buffered saline
IC50	Half maximal inhibitory concentration
