# Supplementary material for: Preliminary Assessment of the Mucosal Toxicity of Tea Tree (Melaleuca alternifolia) and Rosemary (Rosmarinus officinalis) Essential Oils on Novel Porcine Uterus Models
Source: Int J Mol Sci. 2020 May 9;21(9):3350. doi: 10.3390/ijms21093350 (PMC7247571; doi:10.3390/ijms21093350)
Supplement: Supplementary file 1 [file ijms-21-03350-s001.pdf]

Preliminary Assessment of the Mucosal Toxicity of Tea Tree (*Melaleuca alternifolia*) and Rosemary (*Rosmarinus officinalis*) Essential Oils on Novel Porcine Uterus Models

Martina Bertocchi, Antonella Rigillo, Alberto Elmi, Domenico Ventrella, Camilla Aniballi, Diana G. Scorpio, Maurizio Scozzoli, Giuliano Bettini, Monica Forni and Maria Laura Bacci

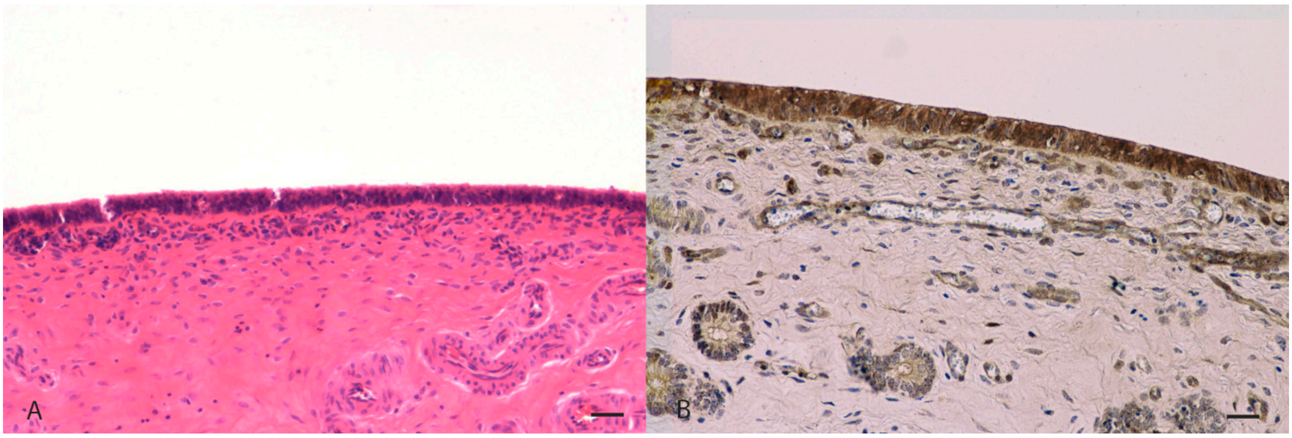

Figure S1. Histologic appearance (A) and immunohistochemical expression of ZO-1 (B) of negative control tissues of porcine uterine mucosa incubated only with emulsifiers and SFM extenders. 200x. Bar = 50 μm.
